# ADHD and depression: investigating a causal explanation

**DOI:** 10.1017/S0033291720000665

**Published:** 2021-08

**Authors:** Lucy Riglin, Beate Leppert, Christina Dardani, Ajay K. Thapar, Frances Rice, Michael C. O'Donovan, George Davey Smith, Evie Stergiakouli, Kate Tilling, Anita Thapar

**Affiliations:** 1 Division of Psychological Medicine and Clinical Neurosciences, MRC Centre for Neuropsychiatric Genetics and Genomics, Cardiff University, Cardiff, UK; 2 Population Health Sciences, Bristol Medical School and MRC Integrative Epidemiology Unit, University of Bristol, Bristol, UK; 3 Centre of Academic Mental Health, Bristol Medical School, University of Bristol, Bristol, UK; 4 Oral and Dental Sciences, University of Bristol, Bristol, UK

**Keywords:** ADHD, ALSPAC, causal, depression, longitudinal, Mendelian randomization

## Abstract

**Background:**

Attention-deficit hyperactivity disorder (ADHD) is associated with later depression and there is considerable genetic overlap between them. This study investigated if ADHD and ADHD genetic liability are causally related to depression using two different methods.

**Methods:**

First, a longitudinal population cohort design was used to assess the association between childhood ADHD (age 7 years) and recurrent depression in young-adulthood (age 18–25 years) in *N* = 8310 individuals in the Avon Longitudinal Study of Parents and Children (ALSPAC). Second, two-sample Mendelian randomization (MR) analyses examined relationships between genetic liability for ADHD and depression utilising published Genome-Wide Association Study (GWAS) data.

**Results:**

Childhood ADHD was associated with an increased risk of recurrent depression in young-adulthood (OR 1.35, 95% CI 1.05–1.73). MR analyses suggested a causal effect of ADHD genetic liability on major depression (OR 1.21, 95% CI 1.12–1.31). MR findings using a broader definition of depression differed, showing a weak influence on depression (OR 1.07, 95% CI 1.02–1.13).

**Conclusions:**

Our findings suggest that ADHD increases the risk of depression later in life and are consistent with a causal effect of ADHD genetic liability on subsequent major depression. However, findings were different for more broadly defined depression.

Attention-deficit hyperactivity disorder (ADHD) is a common, childhood-onset neurodevelopmental disorder that frequently co-occurs with other neuropsychiatric disorders (Jensen & Steinhausen, 2015; Thapar & Cooper, 2016). There is growing recognition that ADHD is commonly associated with depression, which typically onsets later, with an estimated increase in risk for depression of 6.5-fold within the first year of ADHD diagnosis (Gundel, Pedersen, Munk-Olsen, & Dalsgaard, 2018).

One explanation is that ADHD causes depression. For example, ADHD could elicit stressful environmental exposures such as discordant relationships (Harold et al., 2013; Lifford, Harold, & Thapar, 2009), peer-victimization (Schoeler et al., 2019; Sonuga-Barke & Taylor, 2015) and poor academic attainment (Loe & Feldman, 2007), which in turn increase risk of depression (Schoeler, Duncan, Cecil, Ploubidis, & Pingault, 2018; Thapar, Collishaw, Pine, & Thapar, 2012). A causal explanation implies that treatment of ADHD will reduce risk of depression. Consistent with this, a quasi-experimental national registry study found that individuals with ADHD had a 20% lower rate of depression when receiving ADHD medication compared to when they were not, and suggested that ADHD medication was associated with a decreased long-term risk of depression (Chang, D'Onofrio, Quinn, Lichtenstein, & Larsson, 2016).

Twin studies, in contrast, suggest that ADHD and depression co-occurrence is largely explained by shared genetic factors (approximately 70%: Faraone & Larsson, 2019). Also, a recent Genome-Wide Association Study (GWAS) of ADHD reported extensive genetic correlation with other psychiatric disorders, the strongest of which was with depression (major depressive disorder *r*_g_ = 0.42, depressive symptoms *r*_g_ = 0.45) (Demontis et al., 2019). Genetic overlap could mean that alleles manifest as different disorders, either in different people (contributing to ADHD in one person and depression in another) or at different times. For example, the same genetic risks could manifest as ADHD in early-life and depression later: a type of heterotypic continuity (Rutter, Kim-Cohen, & Maughan, 2006). Alternative explanations which would inflate genetic overlap estimates include overlapping symptoms that contribute to each phenotype (e.g. concentration problems) or unmeasured confounders.

Distinguishing between these explanations is important because they have different prevention and treatment implications. Here, we investigated the relationship between ADHD and depression using two study designs (Gage, Munafo, & Davey Smith, 2016; Thapar & Rutter, 2019). The rationale was that if both designs showed evidence in favour of an effect of ADHD on depression, this would strengthen the conclusion that ADHD causes later depression (Lawlor, Tilling, & Davey Smith, 2016). First, to examine whether ADHD preceded depression, we directly examined the association between ADHD in childhood and later depression in young-adulthood in a longitudinal population-based cohort. If childhood ADHD has a potentially causal effect on depression, we would expect to see higher levels of young-adult depression in those with versus without childhood ADHD. Prospective designs reduce the problem of reverse causation although longitudinal association can arise from unmeasured confounding including shared risk factors. Thus, we also used two-sample Mendelian randomization (MR) as an alternative approach to causal inference because provided certain assumptions are met, it is less likely to be affected by confounding (Davey Smith & Hemani, 2014). MR tests whether genetic liability to ADHD causes depression when shared pleiotropic effects between ADHD and depression are taken into account. Genetic instruments were selected from large GWAS data as proxies for exposure (ADHD) and outcome (depression).

## Method

### Longitudinal analyses

#### Sample

Mental health data were used from the Avon Longitudinal Study of Parents and Children (ALSPAC), a well-established prospective, longitudinal birth cohort study. Details of this study are provided in the online Supplementary Material. Individuals were included in our analyses when data were available on ADHD in childhood (see below): *N* = 8310 (56% of those alive at 1 year).

#### ADHD (exposure)

ADHD in childhood was measured using the well-validated, five-item parent-rated ADHD subscale of the Strengths and Difficulties Questionnaire (SDQ) (Goodman, 1997) (range 0–10) at age 81 months (roughly age 7 years) (online Supplementary Table S1a). Individuals were coded as having ‘ADHD’ if they met the >7 recommended cut-point (Goodman, 1997). Total ADHD scores were also computed. Sensitivity analyses were conducted using ADHD diagnosis and more broadly defined ADHD (high symptoms at ages 7, 8, 9 or 11 years) (online Supplementary Material page 3). To investigate the extent to which observed ADHD–depression association could be due to the persistence of ADHD symptoms into young-adulthood, we assessed ‘*adult ADHD*’ using self-reported SDQ ADHD data at age 25 years. There is no research to guide appropriate cut-points for adult ADHD. In line with DSM-5 guidance that fewer symptoms are needed for an ADHD diagnosis in adulthood, we used one point less for the cut-point (the recommended ADHD cut-point for SDQ self-reports is >6, so we used >5 for adult self-reports). Individuals were coded as having ADHD that persists in adult life if they met the relevant cut-points at ages 7 and 25.

#### Depression (outcome)

Depression was measured at five time-points from age 18 years (in young-adulthood), using the 13-item self-rated Short Moods and Feeling Questionnaire (sMFQ) (Angold et al., 1995) (range 0–26): at ages 18, 21, 22, 23 and 25 years (online Supplementary Table S1b). The sMFQ is a well-validated, widely used measure which has been shown to be a valid diagnostic tool for detecting major depressive disorder in community samples (Angold et al., 1995; Thapar & McGuffin, 1998; Turner, Joinson, Peters, Wiles, & Lewis, 2014). We focused on depression after age 18 years as our outcome in the longitudinal analyses to obtain similarity to the depression phenotype in the GWAS from which we derived our instruments for MR (predominantly participants aged ⩾18 years; see MR analyses).

Single episodes of depression are common (Mojtabai, Olfson, & Han, 2016) and depression is highly heterogeneous. For example, depression can be defined broadly (e.g. self-reported responses to one or two questions) as well as more strictly (e.g. DSM-defined major depression or recurrent depression). Recent findings in pre-peer-reviewed format (Cai et al., 2019) suggest that the genetic architecture of broad depression (e.g. from UK biobank; Howard et al., 2019) is not the same as more narrowly defined depression (e.g. recurrent female depression; Converge consortium, 2015). Twin and family studies also suggest that narrowly defined depression is more familial and heritable (Glowinski, Madden, Bucholz, Lynskey, & Heath, 2003; Klein, Shankman, Lewinsohn, Rohde, & Seeley, 2004). In the present analysis, we therefore selected the more narrow outcome of *recurrent depression in young-adulthood* for those who met the recommended >11 sMFQ cut-point (Thabrew, Stasiak, Bavin, Frampton, & Merry, 2018) at least twice in adulthood (⩾18 years) to approximate the depression outcome for the GWAS used in our initial MR analysis (see below). Single time-point total depressive symptom scores were also computed. To investigate whether the observed association between ADHD and depression in young-adulthood could be due to the persistence of childhood depression, we used previous self-reports and classified ‘*childhood depression*’ based on meeting the sMFQ cut-point at ages 10 or 12 years.

#### Covariates

Sex, early social adversity (parent home ownership and crowding during pregnancy), maternal education and maternal prenatal depression were included as potential confounders for the longitudinal analyses.

#### Analyses

There were 8310 individuals with ADHD data at age 7, of these 57% (*N* = 4771) had depression data for at least one time point (full details in online Supplementary Material). Primary analyses were conducted using multiple imputation (MI) by chained equations, including all those with ADHD data (*N* = 8310) to minimize bias from missing depression data (Lawlor et al., 2016; White, Royston, & Wood, 2011). Sensitivity analyses were conducted using MI for the full ALSPAC dataset (i.e. including those without age 7 ADHD data), using inverse probability weighting (IPW) (Seaman & White, 2013) and using listwise deletion to investigate the impact of missing data. Full details of the imputation and IPW models are given in the online Supplementary Material.

### Mendelian randomization

#### Genetic data

For primary analyses examining the effect of ADHD genetic liability on depression, genetic data were used from GWAS of individuals with European ancestry for ADHD (*N* = 53 293: Demontis et al., 2019) and major depression (MD) (*N* = 138 884: Wray et al., 2018, excluding 23andMe and UK Biobank, described in Howard et al., 2019). These are described in more detail in the online Supplementary Material.

Additional analyses were conducted using a larger, more recent depression GWAS (*N* = 500 199: Howard et al., 2019) which included a ‘broad depression’ phenotype based on self-reported help-seeking behaviour for nerves, anxiety, tension or depression (*N* = 361 315: 72% of the GWAS sample).

Previously reported genetic correlations with ADHD are *r*_g_ = 0.42 (s.e. = 0.03) for MD (Demontis et al., 2019) and *r*_g_ = 0.39 (s.e. = 0.10) for broad depression (Howard et al., 2019).

#### Analyses

Two-sample MR utilizes genetic variants from GWAS as instrumental variables (proxies for the exposure). These genetic proxies should satisfy three main assumptions: (i) strong association with the exposure, (ii) no association with confounders of the exposure–outcome association, (iii) association with the outcome exclusively via the exposure (Bowden et al., 2016). Since genetic variants are randomly assorted at meiosis and fixed at conception, this method is more robust to confounding than conventional observational studies. Variants therefore differ regarding exposure and suffer from little confounding, providing some analogy with randomized control trial groups.

Genetic variants (single-nucleotide polymorphisms, SNPs) were extracted from the relevant GWAS using a *p* value threshold of 5 × 10^−08^. SNPs were pruned to be in linkage equilibrium (*r*^2^ < 0.001). Where SNPs were missing in the outcome GWAS, linkage disequilibrium proxies (*r*^2^ = 0.8 within 250 kb) were used. Data were harmonized across the exposure/outcome datasets and palindromic SNPs were excluded: details of the included SNPs are shown in online Supplementary Tables S2 and S3.

Three MR approaches were conducted to estimate the exposure–outcome effect because these have different assumptions: inverse-variance weighted (IVW) linear regression, a weighted median approach and MR-Egger regression. IVW assumes that all instruments are valid and that there is no horizontal pleiotropy (i.e. SNPs are associated with the outcome exclusively via the exposure). The weighted median method estimates the median (rather than the mean) causal estimate and is therefore robust to 50% of the genetic variants being invalid. Finally, MR-Egger regression relaxes the third assumption (i.e. no horizontal pleiotropy), by allowing the intercept of the SNP–exposure and SNP–outcome regression to be non-zero; the intercept thus provides an estimate of horizontal pleiotropy and the slope provides an estimate of the causal effect of an exposure on an outcome accounting for pleiotropic effects. MR-Egger regression is therefore less well powered but is robust when 100% of genetic variants have pleiotropic effects, conditional on the assumption that the pleiotropic effects are distributed independently of instrument strength (instrument strength independent of direct effect; InSIDE) (Bowden et al., 2016) (and the assumption that the instruments are measured without error: NOME assumption).

Instrument strength, heterogeneity and outliers: for IVW analyses, instrument strength was assessed using the *F* statistic (*F* < 10 suggests results may suffer from weak instrument bias) and heterogeneity in the SNP–exposure/SNP–outcome association using Cochrane's *Q* (Bowden et al., 2016). For MR-Egger analyses, heterogeneity was assessed by *I*^2^_GX_ values (*I*^2^_GX_ < 90% suggests a potential violation of the NOME assumption): simulation extrapolation (SIMEX)-adjusted MR-Egger was used to provide bias-adjusted estimates when appropriate (Bowden et al., 2016). Radial plots were used to check for potential outliers (individual variants) between IVW and MR-Egger estimates (Bowden et al., 2018).

Sensitivity analyses were conducted using Steiger filtering to determine the direction of causal effect for each SNP, by checking, for example, that ADHD SNPs explain more variation in ADHD than depression (Hemani, Tilling, & Davey Smith, 2017). In-line with recommendations, we used bidirectional MR to assess reverse causation (the effect of depression genetic liability on ADHD) by repeating analyses with depression genetic variants as the exposure and ADHD genetic variants as the outcome (Davey Smith & Hemani, 2014). MR analyses were conducted using the TwoSampleMR package version 0.4.17 for R.

## Results

### Longitudinal analyses: association between childhood ADHD and adult depression

Of those included in our sample, 6.4% (*N* = 530) scored above the ADHD cut-point in childhood (age 7) and 26.9% had recurrent depression in young-adulthood (high depression symptoms at least twice in young-adulthood). Table 1 shows current rates of depression at each assessment in young-adulthood, as well as demographic information for those with and without childhood ADHD. Approximately 32.7% of those with childhood ADHD had recurrent depression compared to 26.5% of those without ADHD. Conversely, of those with recurrent depression in young-adulthood, approximately 7.8% had childhood ADHD compared to 5.9% in those without.

**Table 1.** Demographic information and prevalence of depression in adult life assessed using the sMFQ in those with and without ADHD in ALSPAC

	With ADHD (*N* = 530)	Without ADHD (*N* = 7780)
Demographics		
Age (months)^a^	81.54 (81.44–81.65)	81.44 (81.41–81.47)
Female sex	31.11% (27.32–35.21)	49.83% (48.72–50.94)
Birth weight (kg)	3.34 (3.30–3.39)	3.44 (3.43–3.45)
Maternal age at childbirth (years)	28.36 (27.94–28.78)	28.98 (28.88–29.08)
Maternal education: university degree	10.69% (8.04–13.34)	15.60% (14.79–16.42)
Parental home ownership	73.86% (70.00–77.71)	83.56% (82.71–84.41)
Depression		
Age 18	25.86% (20.31–31.41)	21.32% (19.98–22.66)
Age 21	21.25% (16.13–26.38)	15.88% (14.60–17.16)
Age 22	21.71% (16.83–26.59)	17.60% (16.36–18.83)
Age 23	29.10% (23.59–34.62)	22.88% (21.53–24.23)
Age 25	27.84% (22.47–33.21)	22.45% (21.13–23.76)
Recurrent depression	32.72% (27.37–38.05)	26.47% (25.15–27.80)

sMFQ, short Moods and Feelings Questionnaire; ADHD, attention-deficit/hyperactivity disorder at age 7 years; ALSPAC, the Avon Longitudinal Study of Parents and Children.

95% confidence intervals in parentheses.

a Age at childhood ADHD assessments, *N* = 8266.

ADHD in childhood was associated with an increased risk of having recurrent depression in young-adulthood: OR 1.35, 95% CI 1.05–1.73, *p* = 0.02. This was robust to controlling for sex, early adversity, maternal education and maternal depression (OR 1.38, 95% CI 1.07–1.79, *p* = 0.01).

ADHD defined as a continuous trait (total symptom scores) was also associated with later recurrent depression (OR 1.06, 95% CI 1.03–1.09, *p* = 1 × 10^−05^). The association with recurrent depression was similar when ADHD was defined as ADHD diagnosis and more broadly defined ADHD (see online Supplementary Material page 3). Finally, investigating associations with continuous depressive symptom scores at single time-points found childhood ADHD to be associated with higher depression symptoms in young-adulthood, but the evidence was weaker than when investigating association with recurrent depression (online Supplementary Table S4).

Sensitivity analyses to assess the impact of missing ADHD data using MI for the full ALSPAC sample (i.e. including those without age 7 ADHD data) and using IPW found a similar pattern of results although with much wider confidence intervals when using IPW (see online Supplementary Material). Analyses using listwise deletion did not find evidence of an association between childhood ADHD and recurrent depression in young-adulthood, likely reflecting bias due to non-random attrition, although confidence intervals were wide and overlapped those from the MI analyses (see online Supplementary Material page 2 and Supplementary Table S5).

#### The contribution of childhood depression

Approximately 7.5% of the sample had childhood depression (15.5% of those with childhood ADHD and 15.2% of those with recurrent depression in young-adulthood). Childhood ADHD was associated with an increased likelihood of recurrent depression in young-adulthood for those *without* childhood depression (OR 1.28, 95% CI 0.96–1.71, *p* = 0.09). There was no evidence that ADHD conferred an additional risk for young-adult depression in those who already had depression in childhood (OR 0.88, 95% CI 0.48–1.62, *p* = 0.69), although the sample size was small.

#### The contribution of adult ADHD

Finally, approximately 2.2% of the sample had adult (persistent) ADHD (34.1% of those with childhood ADHD). Those with adult ADHD had a higher proportion of recurrent depression in young-adulthood compared to those with childhood ADHD only (47.5% compared to 26.4%): OR 2.69, 95% CI 1.61–4.50, *p* = 2 × 10^−04^. The association with later recurrent depression for those whose childhood ADHD did not persist to adulthood was greatly attenuated: OR 0.93, 95% CI 0.67–1.29, *p* = 0.66.

Conversely, those with recurrent depression in young-adulthood were also at an increased risk of having adult ADHD (3.4% compared to 1.6% in those without recurrent depression in young-adulthood): OR 2.51, 95% CI 1.68–3.75, *p* = 8 × 10^−06^.

### Mendelian randomization

MR results are summarized in Table 2. We did not find evidence of weak instrument bias for IVW analyses (ADHD *F* = 31.13, MD *F* = 34.28). MR-Egger indicated potential violation of the NOME assumption (ADHD *I*^2^_GX_ = 38%) so SIMEX-adjusted MR-Egger regression were conducted as a sensitivity analysis.

**Table 2.** Mendelian randomization (MR) results

			IVW (slope)			Weighted median (slope)			MR Egger (SIMEX): slope			MR Egger (SIMEX): intercept		
Exposure	Outcome	SNPs	OR	(95% CI)	*p*	OR	(95% CI)	*p*	OR	(95% CI)	*p*	OR	(95% CI)	*p*
ADHD	Major dep.	10	1.21	(1.12–1.31)	1 × 10^−06^	1.20	(1.08–1.33)	6 × 10^−04^	1.22	(1.13–1.31)	1 × 10^−03^	1.00	1.00–1.01	0.25
Major dep.	ADHD	3^a^	1.80	(0.85–3.82)	0.12	1.26	(0.85–1.86)	0.25	^a^	^a^	^a^	^a^	^a^	^a^
ADHD	Broad dep.	10	1.06	(0.98–1.15)	0.14	1.01	(0.96–1.07)	0.61	1.07	(1.02–1.13)	0.02	1.01	1.01–1.01	2 × 10^−03^
Broad Dep.	ADHD	40	1.79	(1.36–2.36)	3 × 10^−05^	1.65	(1.28–2.12)	1 × 10^−04^	1.76	(1.32–2.34)	5 × 10^−04^	1.00	1.00–1.01	0.30

ADHD, attention-deficit/hyperactivity disorder; Dep, depression; IVW, inverse-variance weighted.

Odds ratios for slope estimates estimate the increase in the odds of the outcome for each unit increase in log-odds genetic liability to the exposure. MR Egger intercept measures horizontal pleiotropy.

a MR Egger (SIMEX) not run due to the small number of SNPs included. No outliers were detected.

For each unit increase in log-odds genetic liability of ADHD, there was evidence consistent with an approximately 21% increased risk in odds of MD, as suggested by the IVW (OR 1.21, 95% CI 1.12–1.31) with very little evidence of heterogeneity (*Q* = 9.22, df = 9, *p* = 0.42). This association was consistent across the different MR approaches (weighted median OR 1.20, 95% CI 1.08–1.33; SIMEX-adjusted MR-Egger OR 1.22, 95% CI 1.13–1.31) and there was very little evidence of pleiotropy (SIMEX-adjusted MR-Egger intercept OR 1.00, *p* = 0.25).

Analyses in the reverse direction (odds of ADHD for each unit increase in log-odds genetic liability of MD) found less consistent evidence of association (IVW OR 1.80, 95% CI 0.85–3.82; weighted median OR 1.26, 95% CI 0.85–1.86) with evidence of heterogeneity (*Q* = 13.50, df = 2, *p* = 1 × 10^−03^) although analyses were limited by the small number of available SNPs for MD (*N* = 3: see Table 2).

Results using GWAS findings for a broader depression phenotype are shown in Table 2. We did not find evidence of weak instrument bias for IVW analyses (broad depression *F* = 37.87). Again, MR-Egger analyses indicated a potential violation of the NOME assumption (ADHD *I*^2^_GX_ = 38%, broad depression *I*^2^_GX_ = 24%) so SIMEX-adjusted MR-Egger regression were conducted.

We found weaker evidence for an impact of ADHD genetic liability on broad depression, consistent with an approximately 7% increase in the odds of broad depression per log odd increase in genetic liability to ADHD, in the presence of horizontal pleiotropy (SIMEX-adjusted MR-Egger slope OR 1.07, 95% CI 1.02–1.13; intercept OR 1.01, *p* = 2 × 10^−03^). Using the broader depression GWAS for MR analysis, association in the reverse direction suggested an approximately 79% increase in the odds of ADHD per log odd increase in genetic liability to broad depression (IVW OR 1.79, 95% CI 1.36–2.36) with evidence of heterogeneity (*Q* = 117.78, df = 39, *p* = 8 × 10^−10^). This was consistent across the different MR approaches (weighted median OR 1.65, 95% CI 1.28–2.12; SIMEX-adjusted MR-Egger OR 1.76, 95% CI 1.32–2.34) with little evidence of horizontal pleiotropy (SIMEX-adjusted MR-Egger intercept OR 1.00, *p* 0.30).

#### Sensitivity analyses

Steiger filtering showed evidence consistent with the causal direction being correct for all the ADHD SNPs. There was evidence that one of the three MD SNPs and 12 of the 40 broad depression SNPs did not meet this criterion (online Supplementary Table S3). Removing these SNPs revealed the same pattern of results as when they were included (online Supplementary Table S6).

## Discussion

This study used two different designs, a longitudinal population cohort design and Mendelian randomization (MR) to investigate a possible causal relationship between ADHD and depression. Previous work has observed association and genetic overlap between ADHD and depression (Demontis et al., 2019) but it is unclear from these studies whether this is due to the same set of risks manifesting as different disorders in different people, at different ages, or because ADHD causes depression (or both). Our longitudinal analyses found evidence in favour of an association between childhood ADHD and subsequent depression in adult life. Findings of MR analyses were different depending on which depression GWAS was used. Using variants from the GWAS of major depression (MD), we observed a causal effect of ADHD genetic liability on depression. However, when using depression variants identified from a larger GWAS of a broader definition of depression, the findings supported the reverse direction of causality whereby depression genetic liability caused ADHD.

In our population cohort, we found that childhood ADHD was associated with an increased risk of recurrent depression in young-adulthood. This association was held when controlling for sex, adversity, maternal education and maternal depression. These UK cohort findings are consistent with Scandinavian patient registry data which found that children diagnosed with ADHD were approximately six times as likely to have depression within 1 year of ADHD diagnosis and twice as likely within 5 years, compared to children without ADHD (Gundel et al., 2018). Ours is the first study to our knowledge that has used a large prospective population-based sample to examine associations between childhood ADHD and recurrent depression over a decade later, and extends previous work in this sample showing an association between childhood ADHD and adolescent depression (Eyre et al., 2019). Our findings suggest that this association is not driven by childhood depression, but rather that children with ADHD develop subsequent depression. We observed that much of this association was explained by the continuation of ADHD into adult life. This has important implications for those assessing and treating adults with ADHD and for clinicians to be mindful of possible undetected depression in adults with ADHD (in whom we found nearly half had recurrent depression).

The results suggesting that those whose ADHD symptoms persist into adulthood are at particular risk of having depression are consistent with previous work suggesting poorer outcomes at age 18 years for those with persistent compared to remitted ADHD (Agnew-Blais et al., 2018). However, it is important to note that ADHD by itself is not a strong risk factor for adult depression: reducing ADHD symptoms may prevent subsequent depression, but many individuals will develop depression for reasons not explained by the presence of ADHD. For example, while approximately 54% of those with ‘adult ADHD’ had depression in young-adulthood, only 2.6% of those with depression in young-adulthood had adult ADHD. The very high rate of ‘recurrent depression’ by age 25 years is surprising. However, rates of depression in young people have risen in recent years (Mojtabai et al., 2016) and it is possible that this was especially well captured in ALSPAC because the sMFQ was administered so frequently.

While those with ADHD are at increased risk for later depression, causality cannot be inferred by a longitudinal observational design alone. Using two-sample MR, we assessed the influence of ADHD genetic liability, rather than directly assessed ADHD, on depression by using genetic variants as instrumental variables. Findings from the MR analyses were not clear-cut however and depended on which depression GWAS dataset was used. Using data from the GWAS of more narrowly defined MD, we found evidence in support of causal effects of ADHD genetic liability on MD. We found little evidence for an association in the reverse direction, although these analyses were underpowered due to the limited number of available SNPs. The largest GWAS of depression to date utilized a much broader depression phenotype (Howard et al., 2019): MR analyses using instruments from this GWAS (40 SNPs) had increased power to detect effects compared to those for MD (10 SNPs). Findings for broad depression were less consistent than for MD, with only weak evidence of ADHD genetic liability influencing depression but in the presence of horizontal pleiotropy (suggesting that ADHD genetic variants also impact on broad depression via routes independent of ADHD phenotypic manifestations). However, MR analysis in the reverse direction suggested broad depression genetic liability influences ADHD. ADHD precedes the onset of depression, but this finding could reflect parental factors, whereby parental (broad) depression genetic liability impacts on offspring ADHD. An alternative explanation, given that broad depression genetic liability shows overlap with a range of different psychiatric traits, is that it might influence early temperament measures that in turn are causally related to ADHD.

The extent to which genetic findings and MR based on minimally phenotyped broad depression phenotypes can be generalized to more strictly defined depression diagnosis has been contested (Cai et al., 2019). Our findings add to this. The GWAS from which our broad depression SNPs were defined found a genetic correlation of 0.87 between the broader phenotype of self-reported help-seeking behaviour in UK Biobank and the primarily clinically defined major depressive disorder phenotype used in previous work (Howard et al., 2019). This suggests there are shared genetic factors between MD and more broadly defined depression, consistent with twin research (Kendler et al., 2019). However, twin studies have also suggested possible differences in the genetic epidemiology of depression based on differing levels of severity, whereby genetic factors may explain less variance for broader, less severe depression phenotypes (Glowinski et al., 2003). Moreover, recent work in pre-peer-reviewed format suggests that depression GWAS based on ‘minimal phenotyping’ may result in the identification of gene variants that are non-specific to depression, that overlap more with other psychiatric traits and disorders (Cai et al., 2019). This could explain why we observe differences in our MR findings depending on which depression GWAS was used.

Taking the longitudinal and MR findings for MD together in combination with previous quasi-experimental work (Chang et al., 2016), our findings converge in favour of the hypothesis that ADHD influences the risk of later depression. However, until additional, larger GWAS are conducted, caution is needed given the inconsistent MR results using GWAS for broad versus major depression. These findings suggest that effective on-going treatment of ADHD may reduce the risk of future depression in those with ADHD. This might be an especially important consideration in the transition from child and adolescent mental health services to adult services at age 18 when many with ADHD get lost to follow-up (Young et al., 2016) and coincides with an increase in rates of MD.

In triangulating findings between methods, it is important to compare possible biases. For observational studies, the main likely sources of bias are residual confounding, reverse causality, misclassification and non-random missingness (Lawlor et al., 2016). Our phenotypic analyses are unlikely to suffer from reverse causality given the early onset of ADHD. When we investigated the effect of possible confounders, our findings were robust to the inclusion of sex, adversity, maternal education and maternal depression as covariates. However, unmeasured confounding is always a potential problem in observational studies (Thapar & Rutter, 2019). Also, ALSPAC like many longitudinal samples suffers from non-random attrition: individuals at elevated risk of psychopathology are more likely to drop-out of the study (Taylor et al., 2018). We used MI to try to minimize the effect of missingness, although this is only valid if the data are missing at random (missingness is independent of the unobserved missing data, given the variables in the imputation model) and the imputation model is correctly specified.

In contrast, the main source of bias for MR is horizontal pleiotropy (genetic variants for the exposure being related to the outcome via routes other than the exposure) (Lawlor et al., 2016). MR analyses for ADHD and MD did not find strong evidence of horizontal pleiotropy suggesting that this bias is unlikely to be substantial, although these analyses were limited due to the variants available. We did find evidence of horizontal pleiotropy for association with broad depression genetic liability. Other sources of bias include population stratification, which will have been minimized by our use of European ancestry GWAS results, although the lack of diversity with respect to ancestral populations and ethnicity (in both methods) may limit the generalizability of our findings. Another assumption of two-sample MR is that the two samples come from the same underlying population: the ADHD and depression GWAS samples come from different populations in that they primarily use child and adult cases respectively, which may affect the results. Finally, we did not find evidence of weak instrument bias for IVW analyses, which will have been minimized by the use of large GWAS (and would bias towards the null; Lawlor et al., 2016). Future work should examine the consistency of findings across different emerging techniques that use genetic data to test causality (O'Connor & Price, 2018). It is also important to note when comparing methods, that our longitudinal analyses focussed on ADHD at age 7 (with secondary analyses including age 25 ADHD data), whereas MR measures lifetime exposure. The causal effect of ADHD on depression estimated by MR could be driven by the persistence of ADHD across the lifespan, which is consistent with our observation of stronger association with depression for ADHD that persisted into adult life.

Both methods were limited by the measures used. The measure used in sensitivity analyses of adult ADHD is yet to be validated for this age-group: this needs further research given the importance of using consistent measures across time. The measures used in both of our methods also meant that we were unable to investigate whether inattention or hyperactivity–impulsivity symptoms are more likely to lead to later depression.

In conclusion, we used two methods to investigate a possible causal impact of ADHD and ADHD genetic liability on depression. Our longitudinal analyses highlight ADHD as a risk factor for depression in young-adulthood and MR analyses supported a causal effect of ADHD on MD although findings were different depending on the definition of depression. Taken together, clinical follow-up studies, one quasi-experimental design and this MR study and cohort design, converge in suggesting that ADHD may have a causal effect on depression. This suggests that effective treatment of ADHD might help prevent the development of depression for some, especially when ADHD persists into adulthood, but that it is unlikely to consistently prevent the development of depression for all.

## Supplementary material

For supplementary material accompanying this paper visit https://doi.org/10.1017/S0033291720000665. click here to view supplementary material
