# Supplementary material for: ADHD and depression: investigating a causal explanation
Source: Psychol Med. 2020 Apr 6;51(11):1890–7. doi: 10.1017/S0033291720000665 (PMC8381237; doi:10.1017/S0033291720000665)
Supplement: Supplementary file 1 [file S0033291720000665sup001.docx]

**Supplementary Material**

**The Avon Longitudinal Study of Parents and Children (ALSPAC)**

Pregnant women resident in Avon, UK with expected dates of delivery 1st April 1991 to 31st December 1992 were invited to take part in the study. The initial number of pregnancies enrolled is 14,541 (for these at least one questionnaire has been returned or a “Children in Focus” clinic had been attended by 19/07/99). Of these initial pregnancies, there was a total of 14,676 foetuses, resulting in 14,062 (96%) live births and 13,988 children who were alive at 1 year of age. When the oldest children were approximately 7 years of age, an attempt was made to bolster the initial sample with eligible cases who had failed to join the study originally. As a result, the total sample size for data collected after the age of seven is therefore 15,454 pregnancies, resulting in 15,589 foetuses. Of these 14,901 (96%) were alive at 1 year of age. The sample is relatively homogeneous in regard to ethnicity (approximately 98% White) (Fraser et al., 2013). Part of this data was collected using REDCap (https://projectredcap.org/resources/citations/). Ethical approval for the study was obtained from the ALSPAC Law and Ethics Committee and Local Research Ethics Committees. Informed consent for the use of data collected via questionnaires and clinics was obtained from participants following the recommendations of the ALSPAC Ethics and Law Committee at the time. Please note that the study website contains details of all the data that is available through a fully searchable data dictionary and variable search tool: http://www.bristol.ac.uk/alspac/researchers/our-data/. Further details of the study, measures and sample can be found elsewhere (Boyd et al., 2013; Fraser et al., 2013; Northstone et al., 2019). Where families included multiple births, we included the oldest sibling. Individuals were included in our analyses when data were available on ADHD in childhood (see below): N=8310 (56% of those alive at 1 year).

**Genome Wide Association Study (GWAS) data**

The ADHD GWAS ADHD (N=53,293: Demontis et al., 2017) included cases and controls from 11 European and North American cohorts aggregated by the Psychiatric Genomics Consortium (PGC) and one population-based cohort from Denmark collected by the Lundbeck Foundation Initiative for Integrative Psychiatric Research (iPSYCH).

The major depression (MD) GWAS (N=138,884: Wray et al., 2018, excluding 23andMe and UK Biobank, descibed in Howard et al., 2019) included cases and controls from 5 cohorts: one previously aggregated by the PGC including 29 European, North American and Australian studies (Major Depressive Disorder Working Group of the Psychiatric et al., 2013) and four population-based cohorts from the USA, UK, Iceland and Denmark (Genetic Epidemiology Research on Adult Health and Aging, Generation Scotland, deCODE and iPSYCH respectively).

The broad depression GWAS (N=500,199: Howard et al., 2019) included, in addition to samples from the MD GWAS described above (Wray et al., 2018), a “broad depression” phenotype based on self-reported help-seeking behaviour for nerves, anxiety, tension or depression in the population-sample UK Biobank (N=361,315: 72% of the GWAS sample).

**Investigating the impact of missing follow-up data**

Of the N=8310 individuals with childhood ADHD data included in our sample, 57% (N=4771) had depression data for at least one time point in young-adulthood: the number of individuals with depression data at each time-point are shown in Supplementary Figure 1. Primary analyses were conducted using multiple imputation including all those with ADHD data (N=8310) (White, Royston, & Wood, 2011a) to minimise bias in estimates resulting from missing depression data; multiple imputation for the full ALSPAC sample (i.e. including those without ADHD data at age 7 years), inverse probability weighting (Seaman & White, 2013) and analyses using listwise deletion were conducted to investigate the impact of missing follow-up data. Imputation is valid if the imputation models are correctly specified, and the data are missing at random (MAR) - i.e. having incomplete depression data may depend on other observed variables (e.g. sex, maternal depression) but cannot depend on the unobserved depression values - even when the proportion of missingness is large (Madley-Dowd, Hughes, Tilling, & Heron, 2019). IPW is valid if the missingness model is correctly specified, and the data are MAR. These assumptions cannot be verified using the observed data – but comparisons between MI and IPW allow some triangulation of the evidence. Complete case analysis (listwise deletion) is valid if the outcome in the analysis models (here, recurrent depression in young-adulthood) is MAR, conditional on the variables in the analysis model. Thus if those with depression tend to participate less in ALSPAC (Supplementary Table 8) then recurrent depression in young-adulthood may be MAR conditional on earlier measures of depression (so MI and IPW would be potentially valid), but not MAR if conditioned only on the variables in the analysis model (so complete case analysis would not be valid).

These methods are detailed below.

***Multiple imputation***

Multiple imputation by chained equations was used to impute missing depression data (continuous scores) at each time-point whereby variables that predicted depression missingness (Supplementary Table 9) and measures of ADHD and depression at different time-points (Supplementary Tables 10 and 6 respectively), together with variables in the analysis model, were included in an imputation model to generate 250 imputed datasets. This was estimated to be a sufficient number of imputations to ensure that standard errors would not change considerably if the data were imputed again (the recommended 2-stage quadratic rule based on the initial imputation of 250 datasets suggested 51 imputations were needed) (von Hippel, 2018). Estimates were combined across imputed datasets using Rubin’s rules (White, Royston, & Wood, 2011b). Monte Carlo errors were all less than 5% of the standard error and FMI (fraction missing information) values were less than 0.6, suggesting the results would not have varied substantially if the data were imputed again (White et al., 2011b). Sensitivity analyses investigating the consistency of results when using multiple imputation for the full ALSPAC sample found a similar pattern of results (Supplementary Table 9).

***Inverse probability weighting***

Inverse probability weighting (IPW) was used to generate weights derived from a logistic regression analysis of missing depression data for a set of measures assessed in or soon after pregnancy with minimal missingness that were that were associated with missing data (shown in Supplementary Table 9). The Hosmer-Lemeshow test was used assess the fit of the missingness model; results did not indicate poor fit (Hosmer-Lemeshow χ2(10)=12.48, p=0.131). Weights ranged from 1.98 to 57.56. IPW revealed a similar pattern of results although with much larger confidence intervals (Supplementary Table 5).

**Broader and more stringent measures of childhood ADHD**

Sensitivity analyses were conducted using (a) a broader measure of childhood ADHD, and (b) a DSM-IV diagnosis of ADHD.

*Broader definition of ADHD* included individuals who met the >7 recommended cut-point on the SDQ ADHD subscale at ages 7, 8, 9 or 11 years. This captured approximately 12.3% of the sample and showed a similar magnitude of association with recurrent depression in young-adulthood as the primary ADHD measure: OR=1.38, 95% CI=1.14-1.66, p=9x10^-04^.

*DSM-IV ADHD diagnosis* was assessed by computer algorithm using parent reports of the Development Well-Being Assessment (Goodman, Ford, Richards, Gatward, & Meltzer, 2000) at age 7 years. This captured 2.0% of the sample and showed a similar magnitude of association with recurrent depression in young-adulthood, although with wider confidence intervals: OR=1.28, 95% CI=0.76-2.15, p=0.36.

| **Supplementary Table 1a**. Strengths and Difficulties Questionnaire subscale items |
| --- |
| 1. Has been restless, overactive, cannot stay still for long |
| 1. Is constantly fidgeting or squirming |
| 1. Is easily distracted, her concentration wanders |
| 1. Thinks things out before acting *(reverse coded)* |
| 1. Sees tasks through to the end, has good attention span *(reverse coded)* |
| **Supplementary Table 1b**. Short Moods and Feelings Questionnaire items |
| 1. I felt miserable or unhappy |
| 1. I didn’t enjoy anything at all |
| 1. I felt so tired I just sat around and did nothing |
| 1. I was very restless^*^ |
| 1. I felt I was no good anymore |
| 1. I cried a lot |
| 1. I found it hard to think properly or concentrate^*^ |
| 1. I hated myself |
| 1. I felt I was a bad person |
| 1. I felt lonely |
| 1. I thought nobody really loved me |
| 1. I thought I could never be as good as other kids |
| 1. I did everything wrong |
| ^*^Items excluded in analyses shown in Supplementary Table 7. |

| **Supplementary Table 2**. ADHD SNPs included in the Mendelian Randomization analyses | | | | | | | | | | | | |
| --- | --- | --- | --- | --- | --- | --- | --- | --- | --- | --- | --- | --- |
|  |  | Alleles | | ADHD | | | Major Depression | | | Broad Depression | | |
|  |  | Effect | Other | β | SE | p | Β | SE | p | β | SE | p |
| 1. | rs10262192 | A | G | 0.07 | 0.01 | 4x10^-08^ | 0.02 | 0.01 | 0.05 | 0.01 | 4x10^-03^ | 7x10^-04^ |
| 2. | rs1222067 | A | C | -0.08 | 0.02 | 8x10^-07^ | 5x10^-04^ | 0.01 | 0.96 | 5x10^-04^ | 5x10^-03^ | 0.92 |
| 3. | rs12265655 | T | C | 0.09 | 0.02 | 2x10^-08^ | 0.04 | 0.01 | 1x10^-03^ | 0.03 | 0.01 | 5x10^-07^ |
| 4. | rs1427829 | A | G | 0.08 | 0.01 | 1x10^-09^ | 0.02 | 0.01 | 0.06 | 0.01 | 4x10^-03^ | 0.04 |
| 5. | rs212178 | A | G | -0.12 | 0.02 | 1x10^-08^ | -0.02 | 0.01 | 0.22 | -1x10^-03^ | 0.01 | 0.88 |
| 6. | rs28483036 | T | C | -0.07 | 0.01 | 1x10^-06^ | -0.01 | 0.01 | 0.46 | 2x10^-03^ | 4x10^-03^ | 0.69 |
| 7. | rs4858241 | T | G | 0.08 | 0.01 | 8x10^-09^ | 0.01 | 0.01 | 0.31 | 0.01 | 5x10^-03^ | 3x10^-03^ |
| 8. | rs4916723 | A | C | -0.08 | 0.01 | 2x10^-08^ | -8x10^-04^ | 0.01 | 0.93 | 0.01 | 4x10^-03^ | 4x10^-03^ |
| 9. | rs74760947 | A | G | -0.18 | 0.03 | 1x10^-08^ | -0.06 | 0.02 | 0.01 | 3x10^-03^ | 0.01 | 0.78 |
| 10. | rs8039398 | T | C | -0.08 | 0.01 | 3x10^-09^ | -0.02 | 0.01 | 0.01 | -2x10^-03^ | 4x10^-03^ | 0.67 |
| ADHD=attention-deficit/hyperactivity disorder; SNP=single nucleotide polymorphism. | | | | | | | | | | | | |

| **Supplementary Table 3**. Depression SNPs included in the Mendelian Randomization analyses | | | | | | | | | | | | | |  |
| --- | --- | --- | --- | --- | --- | --- | --- | --- | --- | --- | --- | --- | --- | --- |
|  |  | Alleles | |  | Major depression | | | Broad depression | | | ADHD | | | |
|  |  | Effect | Other | β | β | SE | P | Β | SE | p | β | SE | p | |
|  | rs12658032^*^ | A | G |  | 0.05 | 0.01 | 2x10^-08^ |  |  |  | 0.07 | 0.01 | 1x10^-07^ | |
|  | rs1950829 | A | G |  | 0.06 | 0.01 | 6x10^-10^ | 0.03 | 4x10^-03^ | 5x10^-12^ | 0.01 | 0.01 | 0.33 | |
|  | rs2568960 | A | G |  | -0.05 | 0.01 | 1x10^-08^ |  |  |  | -0.01 | 0.01 | 0.43 | |
|  | rs1021363^*^ | A | G |  |  |  |  | 0.03 | 5x10^-03^ | 2x10^-11^ | 0.06 | 0.01 | 2x10^-05^ | |
|  | rs10235664^*^ | T | C |  |  |  |  | 0.03 | 5x10^-03^ | 5x10^-08^ | 0.06 | 0.02 | 2x10^-04^ | |
|  | rs10501696 | A | G |  |  |  |  | 0.03 | 4x10^-03^ | 3x10^-11^ | 0.01 | 0.01 | 0.28 | |
|  | rs10913112 | T | C |  |  |  |  | -0.03 | 5x10^-03^ | 5x10^-09^ | 3x10^-04^ | 0.01 | 0.98 | |
|  | rs13037326^*^ | T | C |  |  |  |  | 0.03 | 5x10^-03^ | 2x10^-10^ | 0.05 | 0.02 | 7x10^-04^ | |
|  | rs1367635^*^ | T | C |  |  |  |  | -0.03 | 4x10^-03^ | 4x10^-09^ | -0.04 | 0.01 | 2x10^-03^ | |
|  | rs150186873 | A | C |  |  |  |  | -0.07 | 0.01 | 5x10^-09^ | -0.05 | 0.04 | 0.22 | |
|  | rs150346963 | T | C |  |  |  |  | 0.03 | 4x10^-03^ | 1x10^-10^ | 0.01 | 0.01 | 0.45 | |
|  | rs17641524^*^ | T | C |  |  |  |  | -0.03 | 0.01 | 2x10^-08^ | 0.04 | 0.02 | 0.01 | |
|  | rs1931388 | A | G |  |  |  |  | 0.03 | 4x10^-03^ | 2x10^-11^ | 0.02 | 0.01 | 0.19 | |
|  | rs198457 | T | C |  |  |  |  | -0.03 | 0.01 | 2x10^-08^ | -0.02 | 0.02 | 0.27 | |
|  | rs2111592 | A | G |  |  |  |  | 0.03 | 5x10^-03^ | 1x10^-08^ | -2x10^-03^ | 0.01 | 0.88 | |
|  | rs2214123 | A | G |  |  |  |  | 0.03 | 5x10^-03^ | 9x10^-09^ | 0.02 | 0.01 | 0.10 | |
|  | rs2232423 | A | G |  |  |  |  | 0.06 | 0.01 | 1x10^-18^ | -0.02 | 0.02 | 0.33 | |
|  | rs2418449 | T | C |  |  |  |  | 0.03 | 5x10^-03^ | 4x10^-09^ | 0.02 | 0.02 | 0.18 | |
|  | rs30266^*^ | A | G |  |  |  |  | 0.04 | 5x10^-03^ | 1x10^-15^ | 0.07 | 0.01 | 4x10^-07^ | |
|  | rs3099439^*^ | T | C |  |  |  |  | -0.02 | 4x10^-03^ | 3x10^-08^ | 0.06 | 0.01 | 7x10^-06^ | |
|  | rs3807865 | A | G |  |  |  |  | 0.03 | 4x10^-03^ | 1x10^-12^ | 0.01 | 0.01 | 0.70 | |
|  | rs4141983 | T | C |  |  |  |  | 0.03 | 5x10^-03^ | 1x10^-08^ | 0.01 | 0.01 | 0.44 | |
|  | rs4799949^*^ | T | C |  |  |  |  | -0.03 | 5x10^-03^ | 1x10^-10^ | -0.03 | 0.01 | 0.03 | |
|  | rs4936275 | T | C |  |  |  |  | 0.03 | 4x10^-03^ | 3x10^-10^ | 0.01 | 0.01 | 0.33 | |
|  | rs508502 | T | C |  |  |  |  | -0.03 | 5x10^-03^ | 4x10^-08^ | 3x10^-03^ | 0.02 | 0.85 | |
|  | rs59082935 | T | C |  |  |  |  | 0.04 | 0.01 | 3x10^-08^ | -0.02 | 0.02 | 0.49 | |
|  | rs59283172 | A | G |  |  |  |  | -0.04 | 0.01 | 2x10^-08^ | -0.04 | 0.02 | 0.10 | |
|  | rs61914045 | A | G |  |  |  |  | 0.03 | 0.01 | 8x10^-09^ | 0.02 | 0.02 | 0.22 | |
|  | rs62535714 | A | G |  |  |  |  | 0.03 | 0.01 | 5x10^-09^ | 0.02 | 0.02 | 0.31 | |
|  | rs66511648^*^ | T | C |  |  |  |  | -0.03 | 5x10^-03^ | 6x10^-10^ | -0.03 | 0.02 | 0.03 | |
|  | rs6656912 | T | C |  |  |  |  | -0.03 | 4x10^-03^ | 7x10^-09^ | 0.01 | 0.01 | 0.31 | |
|  | rs699927 | T | G |  |  |  |  | -0.02 | 4x10^-03^ | 4x10^-08^ | -0.02 | 0.01 | 0.14 | |
|  | rs7152906 | T | C |  |  |  |  | -0.03 | 4x10^-03^ | 2x10^-09^ | -4x10^-03^ | 0.01 | 0.75 | |
|  | rs7241572^*^ | A | G |  |  |  |  | 0.03 | 0.01 | 2x10^-09^ | 0.05 | 0.02 | 5x10^-03^ | |
|  | rs72948506 | A | G |  |  |  |  | 0.03 | 5x10^-03^ | 2x10^-08^ | 0.03 | 0.01 | 0.09 | |
|  | rs7551758 | T | G |  |  |  |  | -0.03 | 4x10^-03^ | 5x10^-11^ | -0.01 | 0.01 | 0.29 | |
|  | rs7725715 | A | G |  |  |  |  | 0.03 | 4x10^-03^ | 2x10^-11^ | 0.01 | 0.01 | 0.58 | |
|  | rs843812 | A | G |  |  |  |  | 0.02 | 4x10^-03^ | 1x10^-08^ | 8x10^-04^ | 0.01 | 0.95 | |
|  | rs9364755^*^ | A | G |  |  |  |  | -0.03 | 0.01 | 3x10^-08^ | -0.05 | 0.02 | 1x10^-03^ | |
|  | rs9529218 | T | C |  |  |  |  | -0.03 | 0.01 | 2x10^-10^ | -0.02 | 0.02 | 0.33 | |
|  | rs9536381 | T | C |  |  |  |  | 0.03 | 5x10^-03^ | 3x10^-08^ | -4x10^-03^ | 0.01 | 0.79 | |
|  | rs9831648^*^ | T | G |  |  |  |  | -0.03 | 0.01 | 2x10^-08^ | -0.04 | 0.02 | 0.01 | |
| ADHD=attention-deficit/hyperactivity disorder; SNP=single nucleotide polymorphism. ^*^Removed in Steiger filtering. | | | | | | | | | | | | | |  |

| **Supplementary Table 4**. Associations between childhood ADHD and continuous measures of depression symptoms in young-adulthood | | | |
| --- | --- | --- | --- |
|  | β | (95% CI) | p |
| Age 18 years | 0.61 | (-0.17, 0.140) | 0.12 |
| Age 21 years | 1.15 | (0.41, 1.89) | 2x10^-03^ |
| Age 22 years | 0.69 | (-0.00, 1.39) | 0.05 |
| Age 23 years | 1.12 | (0.37, 1.87) | 4x10^-03^ |
| Age 25 years | 0.91 | (0.11-1.70) | 0.03 |

| **Supplementary Table 5.** Sensitivity analyses investigating the impact of missing outcome data | | | |
| --- | --- | --- | --- |
|  | Proportion with depression | | Association between  ADHD and depression |
|  | With ADHD | Without ADHD |  |
| Primary analyses:  multiple imputation (N=8310) | 32.7%  (27.4-38.1) | 26.5%  (25.1-27.8) | OR=1.35 (1.05-1.73), p=0.02 |
| Multiple imputation to full sample (N=14,692)^*^ | 35.9% (31.5-40.4) | 28.6% (27.4-29.7) | OR=1.40 (1.15-1.70), p=6x10^-04^ |
| Inverse probability weighting (N=1331) | 29.4% (15.6-28.5) | 24.4% (21.6-27.4) | OR=1.29 (0.58-2.88), p=0.53 |
| Listwise deletion (N=1331) | 23.4% (13.2-37.9) | 25.2%  (22.9-27.6) | OR=0.91 (0.46-1.81), p=0.79 |
| 95% confidence intervals in parentheses. ^*^Includes children alive at 1 year and oldest child where multiple siblings. | | | |

| **Supplementary Table 6.** Mendelian Randomisation (MR) results before and after Steiger filtering | | | | | | | | | | | | | | | | | | | |
| --- | --- | --- | --- | --- | --- | --- | --- | --- | --- | --- | --- | --- | --- | --- | --- | --- | --- | --- | --- |
|  |  |  |  | IVW (slope) | | |  | Weighted median (slope) | | |  | MR Egger (SIMEX): slope | | |  | MR Egger (SIMEX): intercept | | | |
| Exposure | Outcome | SNPs |  | OR | (95% CI) | P |  | OR | (95% CI) | P |  | OR | (95% CI) | p |  | | OR | (95% CI) | p |
| *Original results* | |  |  |  |  |  |  |  |  |  |  |  |  |  |  | |  |  |  |
| Major dep. | ADHD | 3^*^ |  | 1.80 | (0.85-3.82) | 0.12 |  | 1.26 | (0.85-1.86) | 0.25 |  | ^*^ | ^*^ | ^*^ |  | | ^*^ | ^*^ | ^*^ |
| Broad Dep. | ADHD | 40 |  | 1.79 | (1.36-2.36) | 3x10^-05^ |  | 1.65 | (1.28-2.12) | 1x10^-04^ |  | 1.76 | (1.32-2.34) | 5x10^-04^ |  | | 1.00 | 1.00-1.01 | 0.30 |
| *After Steiger filtering* | |  |  |  |  |  |  |  |  |  |  |  |  |  |  | |  |  |  |
| Major dep. | ADHD | 2^*^ |  | 1.25 | (0.88-1.77) | 0.21 |  |  |  |  |  | ^*^ | ^*^ | ^*^ |  | | ^*^ | ^*^ | ^*^ |
| Broad Dep. | ADHD | 28 |  | 1.39 | (1.15-1.68) | 7x10^-04^ |  | 1.56 | (1.20-2.02) | 7x10^-04^ |  | 1.38 | (1.22-1.55) | 1x10^-03^ |  | | 1.00 | 1.00-1.01 | 0.75 |
| ADHD=attention-deficit/hyperactivity disorder, Dep = depression, IVW=inverse-variance weighted. Odds ratios for slope estimates estimate the increase in the odds of the outcome for each unit increase in log-odds genetic liability to the exposure. MR Egger intercept measures horizontal pleiotropy. ^*^Weighted median and MR Egger (SIMEX) not run due to small number of SNPs included. | | | | | | | | | | | | | | | | | | | |

| **Supplementary Table 7.** Associations between childhood ADHD and the short Mood and Feelings Questionnaire in young-adulthood, with and without shared items | | | | | | | | | |
| --- | --- | --- | --- | --- | --- | --- | --- | --- | --- |
|  |  |  | Full scale | | |  | Without restless and concentration items | | |
|  | N |  | B | SE | P |  | B | SE | p |
| Age 18 years | 2819 |  | 0.69 | 0.54 | 0.204 |  | 0.56 | 0.47 | 0.232 |
| Age 21 years | 2811 |  | 1.42 | 0.52 | 0.006 |  | 1.07 | 0.45 | 0.018 |
| Age 22 years | 3156 |  | 0.18 | 0.46 | 0.701 |  | 0.09 | 0.40 | 0.822 |
| Age 23 years | 3176 |  | 1.21 | 0.49 | 0.013 |  | 0.96 | 0.42 | 0.022 |
| Age 25 years | 3195 |  | 0.52 | 0.52 | 0.323 |  | 0.26 | 0.46 | 0.574 |
| Continuous scores used as there is no established cut-point for this measure when items have been excluded. | | | | | | | | | |

| **Supplementary Table 8.** Inspecting depression data missingness: Associations between depression (sMFQ) scores and levels of missing depression data in adulthood | | | | | | |
| --- | --- | --- | --- | --- | --- | --- |
|  | Available depression data | | Number of depression (sMFQ) data-points available  in young-adulthood | | | Ordinal regression |
|  |  |  | None (0 time-points) N=3539 | Partial  (1-4 time-points) N=3440 | Complete  (5 time-points) N=1331 | Association between depression score and with number of depression data-points  (1-5) |
|  | N | (%) | Mean depression score (95% CI)^*^ | | | OR (95% CI) |
| Age 10 years | 5870 | (70.64%) | - | 3.97 (3.84-4.10) | 3.68 (3.49-3.86) | 0.97 (0.96-0.98) |
| Age 12 years | 5416 | (65.17%) | - | 4.02 (3.87-4.17) | 3.91 (3.70-4.12) | 1.00 (0.99-1.02) |
| Age 13 years | 4909 | (59.07%) | - | 5.04 (4.86-5.21) | 4.90 (4.64-5.16) | 1.01 (1.00-1.02) |
| Age 16 years | 4243 | (51.06%) | - | 5.87 (5.64-6.09) | 5.83 (5.53-6.14) | 1.01 (1.00-1.02) |
| Age 17 years | 3611 | (43.45%) | - | 6.65 (6.42-6.88) | 6.05 (5.76-6.35) | 0.98 (0.97-0.99) |
| Age 18 years | 2819 | (33.92%) | - | 6.85 (6.55-7.16) | 6.54 (6.23-6.85) | 1.00 (0.98-1.01) |
| Age 21 years | 2811 | (33.83%) | - | 5.84 (5.56-6.13) | 5.33 (5.04-5.62) | 0.99 (0.98-1.00) |
| Age 22 years | 3156 | (37.98%) | - | 6.43 (6.17-6.70) | 5.83 (5.55-6.11) | 0.98 (0.97-1.00) |
| Age 23 years | 3176 | (38.22%) | - | 7.12 (6.84-7.40) | 6.50 (6.20-6.81) | 0.99 (0.98-1.00) |
| Age 25 years | 3195 | (38.45%) | - | 6.82 (6.53-7.11) | 6.36 (6.02-6.69) | 0.99 (0.98-1.00) |
| sMFQ=short Moods and Feelings Questionnaire. Adult data includes ages 18-25 years (used to define outcome variable for main analyses). ^*^Possible range 0-26; recommended cut-point >11 | | | | | | |

| **Supplementary Table 9.** Inspecting depression data missingness: Associations between measures assessed during and soon-after pregnancy and missing depression data in adulthood | | | | | | |
| --- | --- | --- | --- | --- | --- | --- |
|  | Available exposure data | | Number of depression (sMFQ) data-points available  in young-adulthood | | | Ordinal regression |
|  |  |  | None (0 time-points) N=3539 | Partial  (1-4 time-points) N=3440 | Complete  (5 time-points) N=1331 | Association between exposures and number of depression data-points  (0-5) |
|  | N | (%) | Proportion of exposure (95% CI) | | | OR (95% CI) |
| Child female sex | 8310 | (100%) | 0.33 (0.32-0.35) | 0.56 (0.55-0.58) | 0.69 (0.67-0.72) | 2.96 (2.73-3.20) |
| Home ownership | 7876 | (94.78%) | 0.79 (0.77-0.80) | 0.86 (0.85-0.87) | 0.91 (0.89-0.92) | 1.98 (1.76-2.21) |
| Smoked during pregnancy^*^ | 7997 | (96.23%) | 0.20 (0.19-0.22) | 0.14 (0.13-0.15) | 0.09 (0.08-0.11) | 0.54 (0.48-0.60) |
| Maternal prenatal depression | 8105 | (97.53%) | 0.09 (0.08-0.10) | 0.07 (0.06-0.08) | 0.06 (0.05-0.07) | 0.70 (0.60-0.82) |
|  |  |  | Exposure mean (95% CI) | | | OR (95% CI) |
| Maternal age at delivery | 8308 | (99.98%) | 28.27 (28.11-28.42) | 29.21 (29.06-29.36) | 30.03 (29.80-30.26) | 1.06 (1.1-1.62) |
| Birth weight (kg) | 8209 | (98.78%) | 3.43 (3.42-3.46) | 3.43 (3.42-3.45) | 3.41 (3.38-3.44) | 0.94 (0.87-1.01) |
| Crowding index | 8003 | (96.31%) | 1.92 (1.89-1.95) | 1.72 (1.69-1.75) | 1.60 (1.56-1.64) | 0.74 (0.71-0.77) |
| Parity | 8064 | (97.04%) | 0.87 (0.84-0.90) | 0.76 (0.73-0.79) | 0.69 (0.64-0.73) | 0.87 (0.83-0.90) |
| Maternal highest education | 8087 | (97.32%) | 2.88 (2.84-2.93) | 3.32 (3.27-3.37) | 3.59 (3.53-3.65) | 1.41 (1.36-1.45) |
| sMFQ=short Moods and Feelings Questionnaire. ^*^In last 2 months of pregnancy | | | | | | |

| **Supplementary Table 10.** Inspecting depression data missingness: Associations between SDQ ADHD subscale scores and missing depression data in adulthood | | | | | | |
| --- | --- | --- | --- | --- | --- | --- |
|  | Available ADHD SDQ data | | Number of depression (sMFQ) data-points available  in young-adulthood | | | Ordinal regression |
|  |  |  | None (0 time-points) N=3539 | Partial  (1-4 time-points) N=3440 | Complete  (5 time-points) N=1331 | Association between ADHD score and number of depression data-points  (0-5) |
|  | N | (%) | Mean ADHD score (95% CI)^*^ | | | OR (95% CI) |
| Age 4 years | 7655 | (92.12%) | 4.24 (2.16-4.33) | 3.74 (3.66-3.82) | 3.57 (3.44-3.69) | 0.91 (0.89-0.82) |
| Age 7 years | 8310 | (100%) | 3.75 (3.67-3.83) | 3.20 (3.13-3.28) | 2.91 (2.80-3.03) | 0.89 (0.88-0.91) |
| Age 8 years | 6748 | (81.20%) | 3.73 (3.63-3.83) | 3.15 (3.06-3.23) | 2.76 (2.63-2.88) | 0.89 (0.88-0.91) |
| Age 10 years | 6802 | (81.85%) | 3.31 (3.15-3.36) | 2.77 (2.69-2.84) | 2.38 (2.27-2.49) | 0.88 (0.86-0.90) |
| Age 12 years | 6197 | (74.57%) | 3.26 (3.15-3.36) | 2.61 (2.53-2.69) | 2.17 (2.06-2.27) | 0.86 (0.84-0.87) |
| Age 13 years | 5951 | (71.61%) | 3.42 (3.31-3.52) | 2.77 (2.69-2.85) | 2.28 (2.18-2.39) | 0.85 (0.83-0.87) |
| Age 16 years | 4896 | (58.92%) | 3.05 (2.92-3.18) | 2.53 (2.44-2.61) | 2.04 (1.93-2.14) | 0.85 (0.83-0.87) |
| Age 25 years (self-rated) | 3353 | (40.35%) | 3.66 (2.96-4.36) | 3.14 (3.05-3.24) | 2.94 (2.84-3.05) | 0.95 (0.92-0.98) |
|  | N | (%) | Proportion with ADHD (95% CI) | | | OR (95% CI) |
| Diagnosis (age 7 years) | 7227 | (86.97%) | 0.03 (0.02-0.04) | 0.01 (0.01-0.02) | 0.01 (0.00-0.01) | 0.40 (0.29-0.56) |
| SDQ= Strengths and Difficulties Questionnaire. sMFQ=short Moods and Feelings Questionnaire. *Possible range 0-10; recommended cut-point >7 for parent-rated scores age 4-17 years | | | | | | |

| **Supplementary Figure 1.** Number of individuals with depression data at each time point | |
| --- | --- |
| a) Individuals with depression data at each assessment in young-adulthood | b) Number of young-adulthood time-points individuals had depression data available for |
| 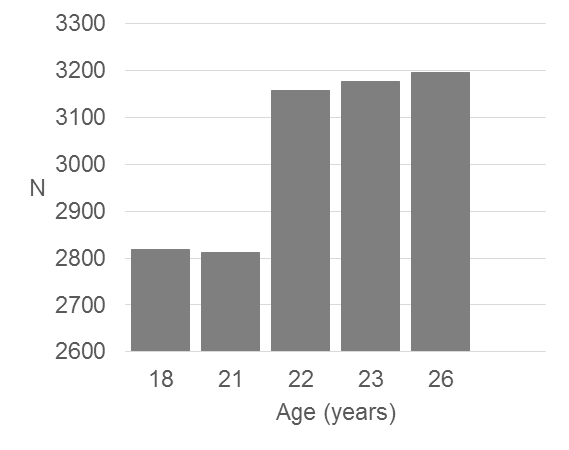 | 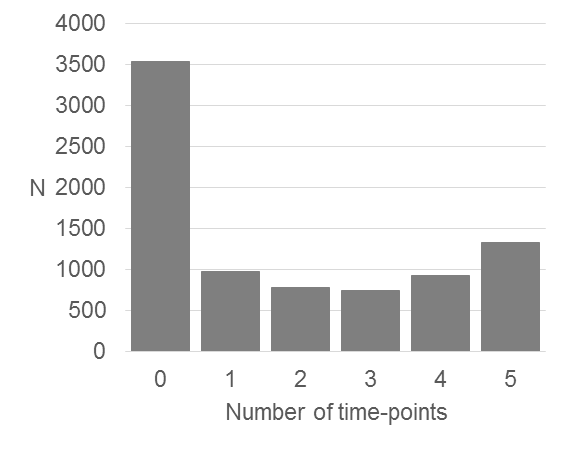 |

**References**

Boyd, A., Golding, J., Macleod, J., Lawlor, D. A., Fraser, A., Henderson, J., . . . Davey Smith, G. (2013). Cohort Profile: the 'children of the 90s'--the index offspring of the Avon Longitudinal Study of Parents and Children. *Int J Epidemiol, 42*(1), 111-127.

Demontis, D., Walters, R. K., Martin, J., Mattheisen, M., Als, T. D., Agerbo, E., . . . Neale, B. M. (2017). Discovery Of The First Genome-Wide Significant Risk Loci For ADHD. *bioRxiv*.

Fraser, A., Macdonald-Wallis, C., Tilling, K., Boyd, A., Golding, J., Davey Smith, G., . . . Lawlor, D. A. (2013). Cohort Profile: the Avon Longitudinal Study of Parents and Children: ALSPAC mothers cohort. *Int J Epidemiol, 42*(1), 97-110.

Goodman, R., Ford, T., Richards, H., Gatward, R., & Meltzer, H. (2000). The Development and Well-Being Assessment: description and initial validation of an integrated assessment of child and adolescent psychopathology. *Journal of child psychology and psychiatry, and allied disciplines, 41*(5), 645-655.

Howard, D. M., Adams, M. J., Clarke, T. K., Hafferty, J. D., Gibson, J., Shirali, M., . . . McIntosh, A. M. (2019). Genome-wide meta-analysis of depression identifies 102 independent variants and highlights the importance of the prefrontal brain regions. *Nature neuroscience, 22*(3), 343-352.

Madley-Dowd, P., Hughes, R., Tilling, K., & Heron, J. (2019). The proportion of missing data should not be used to guide decisions on multiple imputation. *Journal of Clinical Epidemiology, 110*, 63-73.

Major Depressive Disorder Working Group of the Psychiatric, G. C., Ripke, S., Wray, N. R., Lewis, C. M., Hamilton, S. P., Weissman, M. M., . . . Sullivan, P. F. (2013). A mega-analysis of genome-wide association studies for major depressive disorder. *Mol Psychiatry, 18*(4), 497-511.

Northstone, K., Lewcock, M., Groom, A., Boyd, A., Macleod, J., Timpson, N., & Wells, N. (2019). The Avon Longitudinal Study of Parents and Children (ALSPAC): an update on the enrolled sample of index children in 2019. *Wellcome open research, 4*, 51-51.

Seaman, S. R., & White, I. R. (2013). Review of inverse probability weighting for dealing with missing data. *Stat Methods Med Res, 22*(3), 278-295.

von Hippel, P. T. (2018). How Many Imputations Do You Need? A Two-stage Calculation Using a Quadratic Rule. *Sociological Methods & Research*, doi: 10.1177/0049124117747303.

White, I. R., Royston, P., & Wood, A. M. (2011a). Multiple imputation using chained equations: issues and guidance for practice. *Statistics in medicine, 30*(4), 377-399.

White, I. R., Royston, P., & Wood, A. M. (2011b). Multiple imputation using chained equations: Issues and guidance for practice. *Stat Med, 30*(4), 377-399.

Wray, N. R., Ripke, S., Mattheisen, M., Trzaskowski, M., Byrne, E. M., Abdellaoui, A., . . . Major Depressive Disorder Working Group of the Psychiatric Genomics, C. (2018). Genome-wide association analyses identify 44 risk variants and refine the genetic architecture of major depression. *Nat Genet, 50*(5), 668-681.
